# Lifetime risk of maternal near miss morbidity: a novel indicator of maternal health

**DOI:** 10.1093/ije/dyad169

**Published:** 2023-12-18

**Authors:** Ursula Gazeley, Antonino Polizzi, Julio E Romero-Prieto, José Manuel Aburto, Georges Reniers, Veronique Filippi

**Affiliations:** Department of Infectious Disease Epidemiology, London School of Hygiene and Tropical Medicine, London, UK; Department of Population Health, London School of Hygiene and Tropical Medicine, London, UK; Leverhulme Centre for Demographic Science, Nuffield College and Department of Sociology, University of Oxford, Oxford, UK; Department of Population Health, London School of Hygiene and Tropical Medicine, London, UK; Department of Population Health, London School of Hygiene and Tropical Medicine, London, UK; Leverhulme Centre for Demographic Science, Nuffield College and Department of Sociology, University of Oxford, Oxford, UK; Interdisciplinary Centre on Population Dynamics, University of Southern Denmark, Odense, Denmark; Department of Population Health, London School of Hygiene and Tropical Medicine, London, UK; Department of Infectious Disease Epidemiology, London School of Hygiene and Tropical Medicine, London, UK

**Keywords:** Maternal health, maternal near miss, maternal morbidity, maternal mortality, lifetime risk, demographic methods

## Abstract

**Background:**

The lifetime risk of maternal death quantifies the probability that a 15-year-old girl will die of a maternal cause in her reproductive lifetime. Its intuitive appeal means it is a widely used summary measure for advocacy and international comparisons of maternal health. However, relative to mortality, women are at an even higher risk of experiencing life-threatening maternal morbidity called ‘maternal near miss’ (MNM) events—complications so severe that women almost die. As maternal mortality continues to decline, health indicators that include information on both fatal and non-fatal maternal outcomes are required.

**Methods:**

We propose a novel measure—the lifetime risk of MNM—to estimate the cumulative risk that a 15-year-old girl will experience a MNM in her reproductive lifetime, accounting for mortality between the ages 15 and 49 years. We apply the method to the case of Namibia (2019) using estimates of fertility and survival from the United Nations World Population Prospects along with nationally representative data on the MNM ratio.

**Results:**

We estimate a lifetime risk of MNM in Namibia in 2019 of between 1 in 40 and 1 in 35 when age-disaggregated MNM data are used, and 1 in 38 when a summary estimate for ages 15–49 years is used. This compares to a lifetime risk of maternal death of 1 in 142 and yields a lifetime risk of severe maternal outcome (MNM or death) of 1 in 30.

**Conclusions:**

The lifetime risk of MNM is an urgently needed indicator of maternal morbidity because existing measures (the MNM ratio or rate) do not capture the cumulative risk over the reproductive life course, accounting for fertility and mortality levels.

Key Messages

- The global burden of life-threatening maternal near miss (MNM) complications is higher than the burden of maternal death.
- Analogous to the concept of lifetime risk of maternal death (LTR-MD), we propose a new measure of MNM morbidity—labelled the ‘lifetime risk of maternal near miss (LTR-MNM)’—to estimate the cumulative risk of MNM morbidity across the female reproductive life course.
- The LTR-MNM is a novel indicator that estimates the probability that a 15-year-old girl will experience a life-threatening maternal complication during her reproductive lifetime.
- The LTR-MNM is needed because no existing measure of MNM morbidity prevalence (ratio or rate) estimates the cumulative risk over the reproductive age range, accounting for repeated exposures (fertility levels) and background mortality.
- There is utility in comparing trends in the lifetime risk of MNM morbidity alongside trends in the lifetime risk of maternal mortality to better understand changing dynamics of maternal health.

## Introduction

The lifetime risk of maternal death (LTR-MD) is a widely used summary measure of maternal health. As most commonly measured, this denotes the probability of that 15-year-old girl will die from a maternal cause in her reproductive lifetime, accounting for other competing causes of mortality.^1^ Its intuitive appeal means it is used to compare differences between countries and changes over time in World Health Organization (WHO) and United Nations agency joint maternal mortality estimates.^2^ However, maternal deaths are just the tip of the iceberg of poor maternal health outcomes. For every woman who dies from a maternal cause, as many as 20 women may experience a life-threatening ‘maternal near miss’ (MNM) complication,^3^ defined as a ‘woman who nearly died but survived a complication that occurred during pregnancy, childbirth, or within 42 days of termination of pregnancy’.^4^ For the WHO definition, cases are identified based on clinical, laboratory and management-based criteria of organ dysfunction; these criteria are selected such that women would die without emergency care in hospitals.^4^

Substantial reductions in maternal mortality have occurred in the last two decades^2^ as countries advance through the obstetric transition—the secular shift from high to low maternal mortality and direct obstetric to indirect causes of maternal death.^5^ Expansions in access to and improvements in the quality of emergency obstetric care mean that many more women who experience a life-threatening complication now survive pregnancy and the immediate 42-day post-partum period.^5^ The ratio of MNM cases to maternal deaths can be interpreted as a measure of the quality of obstetric care: the higher the ratio, the better the capacity of a health system to manage obstetric emergencies.^4^ Nonetheless, experiencing a complication of this severity may have significant sequelae far beyond 42 days post-partum, including for women’s long-term survival, physical and mental health outcomes, and ability to perform economic and social functions.^3^^,^^6–11^ Given the substantial relative contribution of maternal morbidity to adverse pregnancy outcomes, better indicators are needed for maternal health monitoring and advocacy.

Analogous to the concept of LTR-MD, we propose a new indicator—the lifetime risk of MNM (LTR-MNM)—to measure the probability that a 15-year-old girl will experience a life-threatening MNM complication during her reproductive lifetime. This novel metric is required because existing measures of the frequency of MNM in relation to either the number of live births (MNMRatio) or the female population of reproductive age (MNMRate) do not quantify the cumulative risk of maternal morbidity over a woman’s reproductive life from repeated exposures to pregnancy and childbirth. Nor do they capture how the risk of experiencing an MNM during the reproductive life course is dependent upon surviving from ages 15 to 49 years (i.e. all-cause mortality levels, including maternal causes). Hence, the significance of introducing this new indicator is the need to move beyond measuring the discrete risk of a near miss event and instead capture the cumulative impact of MNM morbidity across the female reproductive life course. As a function of the MNM ratio, fertility and mortality levels, the LTR-MNM addresses this deficit and captures potentially countervailing dynamics.

Using the equation for the LTR-MD as a starting point, we present two methods for the calculation of the LTR-MNM, the choice of which depends on the availability of age-disaggregated MNM data. We describe the step-by-step calculation of the LTR-MNM for Namibia—a country that has achieved a substantial reduction in maternal mortality since 2000,^2^ but where the burden remains ‘high’ at 223 maternal deaths per 100 000 live births.^2^^,^^5^ The calculation combines the national-level estimate of the MNM ratio from 2019^12^ with fertility and survival data from the United Nations World Population Prospects.^13^ Finally, we discuss the strengths and limitations of our proposed indicator.

## Development of the indicator

To calculate the LTR-MNM, we adapt the established method for calculating the LTR-MD. As described by Wilmoth *et al.*,^1^ the LTR-MD can be calculated by using the Maternal Mortality Ratio (i.e. the number of maternal deaths per 1000 live births) or the Maternal Mortality Rate (i.e. the number of maternal deaths per 1000 woman-years lived) as follows:

(1) LTRMD= ∑xx+nMMRation x · fn x·Ln xl15= ∑xx+nMMRaten x ·Ln xl15  

where fn x is the fertility rate between ages x and x+n (where *n* is the length of the age interval), fn x  =Bn xWn x, Bn x is the number of live births for women aged x to x+n, and Wn x is the number of woman-years of exposure for ages x to x+n, in the observed population; Ln x is the number of woman-years of exposure to the risk of dying from maternal or other causes between ages x and x+n, and $l_{15}$ is the probability that a girl will survive to age 15 years. Both Ln x and $l_{15}$ can be obtained from a female-population life table. To calculate the cumulative risk of maternal death across the female reproductive life course, all values are summed from x to x+n, where x is age 15 years, n is an interval of 35 years, and hence x to x+n denotes age 15 to the end of the 49th year. Using period data, the LTR-MD quantifies the risk of death from a maternal cause in a synthetic cohort, conditional on survival to age 15 years, accounting for competing causes of mortality.

Analogously, the LTR-MNM can be calculated by using either (i) the MNM ratio (MNMRatio: the number of MNMs per 1000 live births) or (ii) the MNM rate (MNMRate: the number of MNMs per 1000 woman-years lived). As the MNMRatio is more frequently available, we use this to calculate the LTR-MNM as follows:

(2) LTRMNM= ∑xx+nMNMRation x ·fn x·Ln xl15  

Equation (2) measures the risk of experiencing an MNM during the reproductive life course, conditional on survival to age 15 years and accounting for mortality between the ages 15 and 49 years. As with the LTR-MD, the LTR-MNM is a population average that accounts for age-specific fertility, and hence a women’s repeated exposure to near miss morbidity, but does not account for parity-specific risks because these data are so rarely available.

Where available, the MNMRatio used to estimate the LTR-MNM should be both nationally representative and population-based. As women with an MNM would likely have died without receiving care at the facility, a facility-based estimate of the numerator of the MNMRatio should closely approximate the true number of cases in a community. However, in settings with low levels of institutional delivery, facility-based estimates of live births are likely to underestimate total births in the community, and hence overestimate the MNMRatio. To better approximate the LTR-MNM, a facility-based MNMRatio estimate can be adjusted using the institutional delivery rate to account for births occurring at home; this adjustment is more accurate when facility-based estimates encompass all levels of care (primary, secondary and tertiary). Caution is advised when interpreting the LTR-MNM in cases in which institutional delivery is low and live birth estimates derive solely from tertiary hospitals. See the Appendix for further details.

Below we describe two methods to calculate the LTR-MNM depending on the availability of age-disaggregated estimates of the MNMRatio. All procedures were conducted using R^14^ and are fully reproducible from open data. Our code is posted in a public code repository, available at doi.org/10.17605/OSF.IO/UYZ5H.

### Calculation when (abridged) age-specific MNM data are available

Where age-specific MNM data are available, estimates of the LTR-MNM should use the age-specific MNMRatio. In practice, as the MNMRatios for single-year age groups are virtually never available, the MNMRatio for 5-year age groups is likely the optimum age-disaggregated near miss data. Calculation of the LTR-MNM by 5-year age groups assumes that the MNMRatio, fertility and survival are constant throughout each 5-year age interval.

To demonstrate the calculation of the LTR-MNM in Namibia in 2019 with abridged MNMRatio data, we used a summary MNMRatio for ages 15–49 years of 8.03 per 1000 live births.^12^ This estimate derives from a national MNM surveillance study in Namibia from 2019 that identified MNM events across all hospitals in the country and live births from the Namibian National Health Information System.^12^ As age-disaggregated data for the MNMRatio were not available for Namibia, we simulated possible age patterns of the MNMRatio by 5-year age intervals as follows: we used an estimate of the number of total births by 5-year age group in Namibia from the United Nations World Population Prospects 2019, adjusted for a stillbirth rate of 17.68 per 1000,^15^ and then simulated possible age distributions of MNM cases, for an observed MNMRatio for ages 15–49 years of 8.03. Following evidence on the MNMRatio by age group from Brazil^16^ and global evidence on the risk of maternal death by age,^17^ we hypothesized that a ‘J-shaped’ risk profile might be most plausible and this was used for the worked example: a slightly higher risk for adolescent ages 15–19 years, falling to a minimum at ages 20–24 years and increasing with maternal age thereafter. Finally, we test the sensitivity of the LTR-MNM to the assumed age pattern of the MNMRatio.

In addition to the MNMRatio, we also used open-access estimates of age-specific fertility rates, fn x, survivors to age 15 years, *l_15_*, and the number of woman-years lived in the interval, Ln x, by 5-year age group from the United Nations World Population Prospects abridged life tables for Namibia in 2019^13^ to calculate the LTR-MNM.

Applying Equation (2), the steps are as follows:

- For each age group, the MNMRatio is multiplied by the age-specific fertility rates, fx n .
- This is then multiplied by Lxn l15, which is the expected number of years lived in the age interval for a girl who survived to her 15th birthday.
- Estimates of the LTR-MNM for each 5-year age group are summed to get the final LTR-MNM.
- Reciprocating this total expresses the LTR-MNM as a risk of 1 in *n*.

### Calculation when only summary estimates of MNM for all ages 15–49 years combined are available

Age-disaggregated MNM estimates—even by 5-year age group—are often not available. Rather, an estimate of the MNMRatio is often calculated for all reproductive ages combined from ages 15 to 49 years. The LTR-MNM can be calculated using this summary estimate, although this assumes that the risk of MNM is constant throughout the reproductive ages. This is a simplifying assumption that is most appropriate for data-scarce contexts or when data aggregation results in a loss of detail. Equation (2) becomes:

(3) LTRMNM= 35 MNMRatio15·∑xx+nLn xl15· fn x 

where  35 MNMRatio15 denotes the summary estimate of the MNMRatio between ages 15 and 49 years (age 15 plus an interval of 35 years). Equation (3) can be further simplified to remove age-specific mortality:

(4) LTRMNM= 35 MNMRatio15·NRR·SRB100+1 · l0l15 

where $l_{0}$ is the initial female-population radix (100 000), NRR is the net reproduction rate and SRB is the sex ratio at birth. As the NRR is expressed in terms of female births only, this must be adjusted using the SRB to account for both male and female births included in the fertility rate, fn x. The observed SRB in Namibia in 2019 was 101 boys to 100 girls,^13^ hence Equation (4) becomes:

(5) LTRMNM= 35 MNMRatio15·NRR·2.01 · l0l15 

Note that, for most countries with a typical SRB of 105 boys to 100 girls, the scaling factor would be 2.05; for countries with high sex selection at birth, it could be much higher.

The steps in this calculation are as follows:

- The summary MNMRatio is multiplied by the NRR and the SRB scaling factor.
- This is then multiplied by $\frac{l_{0}}{l_{15}}$, which is the inverse probability of surviving from birth to age 15 years.
- Reciprocating this total expresses the LTR-MNM as a risk of 1 in *n*.

#### Lifetime risk of severe maternal outcome

The concept of LTR-MNM can be used in addition to the LTR-MD to estimate the lifetime risk of severe maternal outcome (LTR-SMO). As SMO is the summation of MNMs and maternal deaths, the LTR-SMO becomes:

(6) $${\mathrm{LTR}}_{\mathrm{SMO}}=\;{\mathrm{LTR}}_{MD}+{\mathrm{LTR}}_{\mathrm{MNM}}\;$$

#### Uncertainty

Where estimates of the MNMRatio derive from surveys, the LTR-MNM is subject to sampling variability. In frequentist models, the 95% CI for the MNMRatio could be used to calculate corresponding uncertainty in the LTR-MNM. In Bayesian models, an 80% uncertainty interval for the MNMRatio and LTR-MNM could be estimated using the 10th and 90th percentiles of the posterior distribution.^2^

## Application

### Calculation when (abridged) age-specific MNM data are available

Table 1 presents the simulated age-disaggregated MNMRatio data, the United Nations World Population Prospects fertility and survival data, and the calculation of the LTR-MNM by each 5-year age group when a ‘J-shaped’ distribution of MNMRatio was assumed.

**Table 1:** Lifetime risk of maternal near miss in Namibia in 2019: calculation assuming ‘J-shaped’ age distribution of the maternal near miss ratio

Age (years)	MNM cases^a^	Live births^b^	MNMRatio^a^^,^^b^ per 1000	fxn per 1000 women^c^^,^^d^	Ln x ^d^	$l_{15}$ ^d^	Lxn l15	LTR-MNM
15–19	68	7939	8.57	66.9	474 931.6	95 283	4.98	0.0029
20–24	82	18 050	4.57	154.3	470 667.4		4.94	0.0035
25–29	107	18 241	5.86	160.0	464 275.4		4.87	0.0045
30–34	143	12 772	11.19	140.9	455 466.6		4.78	0.0075
35–39	91	7492	12.19	103.9	443 928.0		4.66	0.0059
40–44	44	2895	15.06	45.6	429 401.3		4.51	0.0031
45–49	11	612	17.57	11.2	411 688.2		4.32	0.0008
Total	546	68 001	8.03^e^					0.0282 (2.8%) 1 in 35

a Simulated data.

b Data from United Nations World Population Prospects Namibia 2019 total births,^13^ adjusted by stillbirth rate of 17.68 per 1000.^15^

c Values expressed per 1000 are divided by 1000 before calculation.

d United Nations World Population Prospects Namibia 2019.

e Maternal near miss ratio for ages 15–49 years combined = 546/68 001 = 8.03 per 1000 live births.

LTR-MNM, lifetime risk of maternal near miss; MNM, maternal near miss.

In this application, the resulting LTR-MNM was 1 in 35, such that, conditional upon surviving to age 15 years, a girl will face a 1 in 35 chance of experiencing an MNM complication during her reproductive lifetime, accounting for survival from ages 15 to 49 years. This compares with a LTR-MD of 1 in 142 (see ‘Lifetime risk of severe maternal outcome’, below).

#### Sensitivity of the LTR-MNM estimate to the age pattern of the MNMRatio

**Figure 1:**
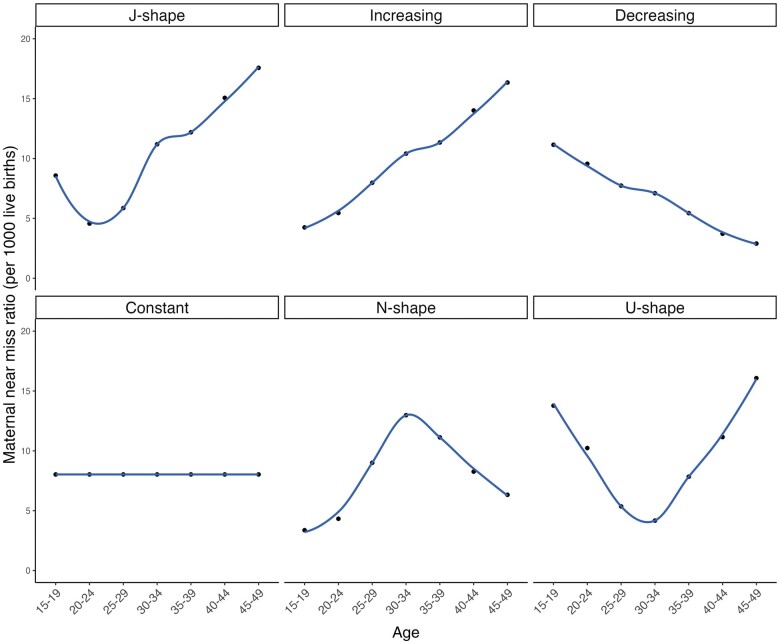
Simulated age distributions of the maternal near miss ratio in Namibia 2019. All distributions have a maternal near miss ratio for ages 15–49 years combined of 8.03 per 1000 live births

For the worked example above, we assumed a ‘J-shaped’ age profile for the MNMRatio. In reality, for a given level of maternal morbidity for reproductive ages 15–49 years combined (8.03 per 1000 live births), the age pattern of the MNMRatio could adopt a variety of shapes (e.g. U-shaped, Increasing, N-shaped, Constant, Decreasing—though N-shaped, Constant and Decreasing are less likely, given what is known about risk of maternal death by age^17^). Figure 1 shows simulated MNM age distributions and Table 2 shows the corresponding estimates of the LTR-MNM. Despite substantial differences in the underlying MNMRatio by age group, the resulting LTR-MNMs are similar. Full calculations for each age distribution can be found in Supplementary Table S1 (available as Supplementary data at *IJE* online).

**Table 2:** Sensitivity of lifetime risk of maternal near miss for Namibia 2019 to the age pattern of the maternal near miss ratio

Age distribution of MNM	LTR-MNM	LTR-MNM 1 in *n*
J-shape	0.0282	1 in 35
N-shape	0.0274	1 in 36
U-shape	0.0261	1 in 38
Constant	0.0262	1 in 38
Decreasing	0.0252	1 in 40
Increasing	0.0278	1 in 36

LTR-MNM, lifetime risk of maternal near miss; MNM, maternal near miss.

### Calculation when only summary estimates of MNM for all ages 15–49 years combined are available

By using the observed NRR of 1.554 (WPP Namibia 2019)^13^ and applying Equation (4), the LTR-MNM becomes:

(7) $${\mathrm{LTR}}_{\mathrm{MNM}}=0.00803\;\cdot 1.554\cdot 2.01\cdot \frac{100\;000}{95\;283}=0.0263\;(2.63\%)\;\mathrm{or}\;1\;\mathrm{in}\;38$$

This summary estimate of the LTR-MNM for ages 15–49 years combined falls within the results for the different possible age distributions above (1 in 40 to 1 in 35), which suggests that Equation (4) is a reasonable approximation where age-disaggregated MNM data are not available.

#### Lifetime risk of severe maternal outcome

Using an estimated MNMRatio of 223 per 100 000 live births for Namibia in 2019,^2^ the LTR-MD is 0.00702 or 1 in 142 (using Equation (4) with the MNMRatio). Using the aggregate estimate of the LTR-MNM (0.0263), Equation (6) for the LTR-SMO becomes:

(8) $${\mathrm{LTR}}_{\mathrm{SMO}}=\;0.00702\;+\;0.0263=\;0.0333\;\left(3.33\%\right)\;\mathrm{or}\;1\;\mathrm{in}\;30$$

This means that, in 2019, there was a 1 in 30 risk that a 15-year-old girl in Namibia would experience either a maternal death or an MNM complication during her reproductive lifetime. MNM morbidity accounts for 79% of the LTR-SMO in this example. The relative contribution of near miss morbidity will vary depending on a country’s position in the obstetric transition.

## Discussion

Life-threatening MNM morbidities are complications so severe that the woman almost died.^4^ Relative to maternal mortality, MNM complications and their sequelae affect many more women, their families, communities and health systems.^18^^,^^19^ As countries progress through the obstetric transition, emergency obstetric care saves more women’s lives after life-threatening complications and the relative contribution of maternal morbidity to maternal (ill)health increases.^5^ This makes MNM an important indicator for advocacy and surveillance, and to identify approaches to improve quality of care.^20^

We propose an extension to the concept of lifetime risk of maternal death (LTR-MD) to MNM morbidity—called the lifetime risk of MNM (LTR-MNM)—to address the deficit of comparable indicators that measure the cumulative impact of maternal morbidity across the female reproductive life course.^11^^,^^21^ The LTR-MNM is a novel indicator to estimate the risk that a 15-year-old girl will experience an MNM complication in her reproductive lifetime. Unlike existing measures of near miss prevalence (e.g. ratio or rate), the LTR-MNM uses fertility rates to account for women’s repeated exposure to the risk of MNM morbidity and adjusts for survival from ages 15 to 49 years. Akin to the LTR-MD, the intuitive appeal of the LTR-MNM may contribute to improved recognition of the global burden of maternal morbidity and strengthen advocacy for its prevention.^22^

Aside from the MNMRatio, the calculation of LTR-MNM uses the same inputs as required for the LTR-MD, increasing the usability of the LTR-MNM. Though the availability of age-disaggregated MNMRatio estimates is often poor, especially in low-resource settings where the burden of maternal morbidity is highest, we have shown that the summary-level estimate of the LTR-MNM falls within the range of estimates derived from age-specific data. If the risk of near miss increases after a certain age, as is the case with the risk of maternal death,^17^ the summary estimate of LTR-MNM may be an underestimate and is therefore best interpreted as a lower bound on the true cumulative risk of near miss morbidity.

There is scope for future research to decompose differences in the LTR-MNM into changes in (i) the risk of near miss associated with each pregnancy (MNMRatio); (ii) the number of times women are exposed (fertility levels, fxn ) and (iii) all-cause mortality (Lxn ). Disentangling these dynamics can improve our understanding of the global burden of maternal morbidity across the female reproductive life course.

### Limitations

As with (period) life expectancy and the LTR-MD, the LTR-MNM is a synthetic cohort measure of population health in which rates observed in a particular year are assumed to be constant for future cohorts. It cannot be interpreted as a prediction of the lifetime risk of an MNM in a real cohort because the MNM, mortality or fertility rates may change in the future. Second, heterogeneity may cause us to over- or underestimate the LTR-MNM: women who experience an MNM may face elevated mortality risks (from maternal and other causes^3^) and therefore have a lower Ln x schedule; they may have either a lower fn x schedule if women delay or limit future childbearing after an initial near miss^23^ or a higher fn x if the near miss coincided with a perinatal death.^8^ Third, experiencing a near miss is a potentially repeating, non-independent event because having an initial near miss may increase a woman’s future risk of experiencing a subsequent near miss. Our calculation does not account for this clustering but amounts to a population average. Finally, estimates of the MNMRatio often derive from surveys of tertiary facilities. These may underestimate live births (thereby overestimating the MNMRatio) even after adjustment using the institutional delivery rate and makes national-level estimation of the LTR-MNM difficult. Further work is required to inform the aggregation of MNM data to produce nationally representative estimates of the LTR-MNM.

## Conclusion

We propose the lifetime risk of MNM as a much-needed new summary measure of maternal health, in addition to mortality. Comparability of estimates would benefit from improvements in the national-level aggregation of MNM, especially in high burden settings.

## Ethics approval

Ethics approval was not required to demonstrate our new indicator as we used population-level data available in the public domain (United Nations World Population Prospects).

## Supplementary Material

dyad169_Supplementary_Data

## Data Availability

All data used in this article are freely available for download from the United Nations World Population Prospects Download Center at https://population.un.org/wpp/Download/Standard/CSV/. All code is available at http://doi.org/10.17605/OSF.IO/UYZ5H.
