# Supplementary material for: Lifetime risk of maternal near miss morbidity: a novel indicator of maternal health
Source: Int J Epidemiol. 2023 Dec 18;53(1):dyad169. doi: 10.1093/ije/dyad169 (PMC11212495; doi:10.1093/ije/dyad169)
Supplement: dyad169_Supplementary_Data [file dyad169_supplementary_data.docx]

**Supplementary Materials**

**Lifetime risk of maternal near miss morbidity: A novel indicator of maternal health**

Contents

[1. Calculation of the lifetime risk of maternal near miss for potential age distributions of the maternal near miss ratio 2](#_Toc150940954)

[2. Bias in the maternal near miss ratio and maternal near miss rate 4](#_Toc150940955)

# Calculation of the lifetime risk of maternal near miss for potential age distributions of the maternal near miss ratio

As shown in Table S1, calculation of the lifetime risk of maternal near miss (LTR-MNM) with age-disaggregated data depends on the age pattern of the maternal near miss ratio (MNMRatio). The LTR-MNM varies from 0.0252 (1 in 40) to 0.0282 (1 in 35). When we assume the MNMRatio is constant across the reproductive ages 15-49, the LTR-MNM is 0.0262 (1 in 38); this estimate falls within the range of the age-disaggregated estimates. Hence, the LTR-MNM using an estimate of the MNMRatio for all ages combined is a reasonable approximation when age-disaggregated data are not available.

Decreasing, Constant, and N-shaped are unlikely based on what we know about the age pattern of maternal mortality. Maternal near miss are expected to be so close to death that we would expect the age pattern to behave similarly.

**Table S1: The lifetime risk of maternal near miss, Namibia 2019 calculation for each simulated maternal near miss age distribution**


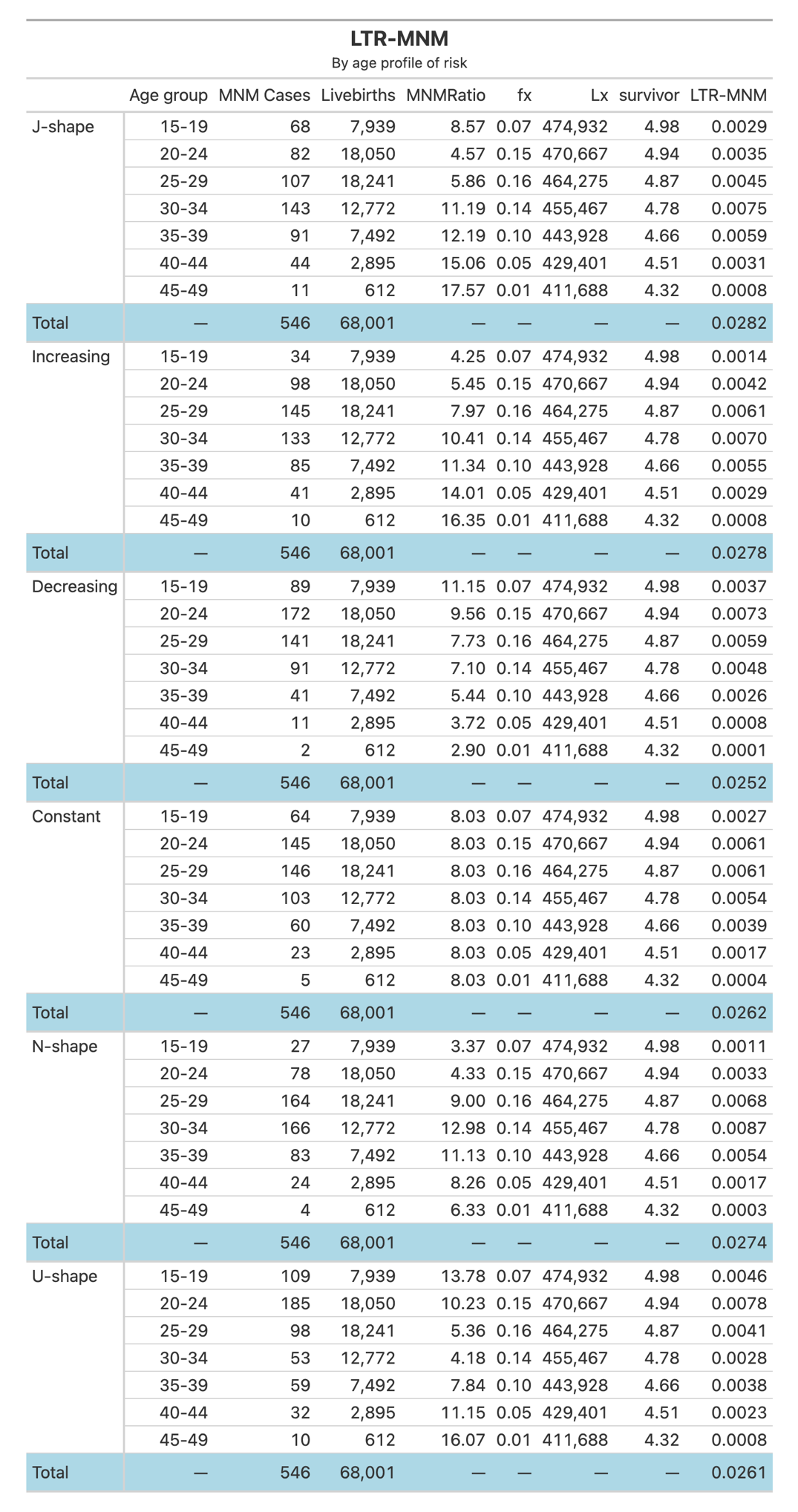


Columns from left to right: Age group denotes five year age group from 15 to 49 years; MNM cases denotes simulated distribution of maternal near miss cases across the five year age group; livebirths denotes the number of live births in five year age group from World Population Prospects (adjusted by stillbirth rate of 17.68 per 1000); MNMRatio denotes the corresponding maternal near miss ratio for simulated distribution of maternal near miss cases; fx denotes fertility rates by five year age group; Lx are the person-years lived in five year age group; survivor denotes the person years divided by number of survivors at age 15 ($\boldsymbol{l}_{\boldsymbol{15}}$ = 95283); LTR-MNM is the lifetime risk of maternal near miss for a given age distribution of near miss cases, for a fixed prevalence of maternal near miss morbidity (8.03 per 1000 live births).

# Bias in the maternal near miss ratio and maternal near miss rate

Where available, the MNMRatio used to estimate the LTR-MNM should be both nationally representative and population-based. As women with a maternal near miss would likely have died without receiving care at the facility, a facility-based estimate of MNM cases should closely approximate the true number of cases in a community. Facility-based estimates of the numerator of the MNMRatio are therefore likely to be representative of MNM in the population. However, the facility-based estimates of the number of live births may be an underestimate of the true number of live births in the community, especially when the prevalence of institutional delivery is low and there are significant numbers of home births. If the denominator is an underestimate, this would result in an upwardly biased estimate of the MNMRatio.

If the MNMRatio is biased, this also results in a biased maternal near miss rate (MNMRate). The following Equations 1-7 show how an unbiased estimate of the MNMRate can be derived from the biased estimate and the number of births occurring within (vs. outside) a facility. This adjusted MNMRate (and hence MNMRatio) can then be used in calculations of the LTR-MNM.

**Starting with the relation between the MNMRate and the MNMRatio**:

$$\left( 1 \right) {MNMRate}_{biased}={{}{}MNMRatio}_{biased} \cdot{}_{n}{f_{x}}$$

This becomes:

$$\left( 2 \right) {MNMRate}_{biased}=\frac{{{}{}All MNM}}{Births in facility} \cdot\frac{All births}{All exposures}$$

Rearranging the terms gives:

$$\left( 3 \right) {MNMRate}_{biased}=\frac{{{}{}All MNM}}{All exposures} \cdot\frac{{{}{}All births}}{Births in facility}$$

Hence, an unbiased estimate of the MNMRate can be derived as follows:

$$\left( 4 \right) \frac{{{}{}All MNM}}{All exposures} = {MNMRate}_{biased}\cdot\frac{{{}{}Births in facility}}{All births}$$

$$\left( 5 \right) {MNMRate}_{true}={MNMRate}_{biased}\cdot institutional delivery rate$$

where the institutional delivery rate is the number births in facilities divided by the total number of births. This accounts for the births occurring at home.

This adjustment is more accurate when facility-based estimates encompass all levels of care (primary, secondary, and tertiary). If estimates of live births derive from tertiary facilities only (e.g., referral or teaching hospitals), then adjusting by the institutional delivery rate will still yield an underestimate of the number of births, since women can give birth in many other types of facility. Therefore, caution is advised when interpreting the LTR-MNM in cases where institutional delivery is low and live birth estimates derive solely from tertiary facilities.
